# ZnO/CuO/M (M = Ag, Au) Hierarchical Nanostructure by Successive Photoreduction Process for Solar Hydrogen Generation

**DOI:** 10.3390/nano8050323

**Published:** 2018-05-12

**Authors:** Jinhyeong Kwon, Hyunmin Cho, Jinwook Jung, Habeom Lee, Sukjoon Hong, Junyeob Yeo, Seungyong Han, Seung Hwan Ko

**Affiliations:** 1 Applied Nano and Thermal Science Lab, Department of Mechanical Engineering, Seoul National University, 1 Gwanak-ro, Gwanak-gu, Seoul 08826, Korea; jhs0909k@snu.ac.kr (J.K.); augustinus310@snu.ac.kr (H.C.); jjw5017@snu.ac.kr (J.J.); habeom.lee@snu.ac.kr (H.L.); 2 Department of Mechanical Engineering, Hanyang University, 55 Hanyangdaehak-ro, Sangnok-gu, Ansan Gyeonggi-do 15588, Korea; sukjoonhong@hanyang.ac.kr; 3 Novel Applied Nano Optics (NANO) Lab, Department of Physics, Kyungpook National University, 80 Daehak-ro, Bukgu, Daegu 41566, Korea; junyeob@knu.ac.kr; 4 Department of Mechanical Engineering, Ajou University, 206 Worldcupro, Yeongtong-gu, Suwon 16499, Korea; 5 Department of Mechanical Engineering/Institute of Advanced Machinery and Design (SNU-IAMD), Seoul National University, Gwanak-ro, Gwanak-gu, Seoul 08826, Korea

**Keywords:** hierarchical nanostructure, photochemical, solar water splitting, photoelectrochemical (PEC) cell, surface plasmon

## Abstract

To date, solar energy generation devices have been widely studied to meet a clean and sustainable energy source. Among them, water splitting photoelectrochemical cell is regarded as a promising energy generation way for splitting water molecules and generating hydrogen by sunlight. While many nanostructured metal oxides are considered as a candidate, most of them have an improper bandgap structure lowering energy transition efficiency. Herein, we introduce a novel wet-based, successive photoreduction process that can improve charge transfer efficiency by surface plasmon effect for a solar-driven water splitting device. The proposed process enables to fabricate ZnO/CuO/Ag or ZnO/CuO/Au hierarchical nanostructure, having an enhanced electrical, optical, photoelectrochemical property. The fabricated hierarchical nanostructures are demonstrated as a photocathode in the photoelectrochemical cell and characterized by using various analytic tools.

## 1. Introduction

Recently, increasing demands for clean and sustainable energy sources have promoted the development of solar-based energy generation devices [1,2]. As a solar energy source, the sunlight-driven water splitting photoelectrochemical (PEC) cell has been intensively explored due to an intuitive working principle and various nanostructured metal oxide candidates such as CuO [3,4], Cu_2_O [5], Fe_2_O_3_ [6,7], TiO_2_ [8,9], WO_3_ [10], and ZnO [11,12]. Nanostructured metal oxides have a unique energy bandgap structure with which they can absorb/emit a specific wavelength. For splitting water molecules efficiently, an energy bandgap position of the metal oxide nanomaterial has to be placed near a water reduction (H^+^/H_2_) or a water oxidation (O_2_/H_2_O) level [13,14].

While most of metal oxide nanomaterials are located in the inappropriate bandgap position for water reduction/oxidation, several efforts are executed to extend absorbable wavelength region through doping or catalytic thin film deposition. The dopant doping or metal nanoparticle/layer deposition is typically conducted by flame spray [15], thermal treatment [16], sputtering [17], chemical vapor deposition (CVD) [18], plasma layer deposition (PLD) [19], atomic layer deposition (ALD) [20], and other vacuum-based techniques. Those methods require controlled conditions including long processing time, high temperature and high-vacuum environment. Most of research groups in the field of solar water splitting use vacuum-based and high-cost processing to aim high-efficiency photoelectrochemical (PEC) cell. To escape from the conventional vacuum deposition-based research trend, low-cost wet chemistry-based routes such as electrospinning [21], spray deposition [22], chemical bath deposition (CBD) [23,24], photoreduction process [25,26], and other solution processing methods were introduced. The wet chemistry based photoreduction process ensures rapid processing, enough surface treatment ability on the metal oxide nanostructures and moderate PEC performance.

In this study, we represent a novel fabrication method for the hierarchical nanostructure by using successive photoreduction process. At first, ZnO nanostructure was synthesized by using hydrothermal growth and used as a scaffold for producing hierarchical nanostructure. ZnO is basically a large energy bandgap (3.3 eV) material, which theoretically absorbs UV wavelengths most effectively [27]. In order to tune energy bandgap level of ZnO, photoreduction process is conducted to make ZnO/CuO hetero junction nanostructure. Unlike ZnO nanowires, nanostructured CuO has a moderate energy bandgap (1.35 eV), which can absorb visible wavelength of light. It is widely adopted as a photocathode in a PEC cell because conduction band position of CuO is slightly higher than water reduction level [28]. Afterward, continuing photoreduction process forms ZnO/CuO/M hierarchical nanostructure by synthesizing metal nanoparticles on the ZnO/CuO hetero nanostructure. At this moment, the synthesized silver/gold nanoparticles work as catalyst points or triggers to occur surface plasmon effect. The fabricated ZnO/CuO/M hierarchical nanostructure shows an enhanced optical and PEC property. Consequently, the successive photoreduction process provides and contribute for the water splitting PEC applications by proposing facile fabrication process under wet environments.

## 2. Experimental Section

**Synthesis of ZnO Nanowire Arrays**—Prior to hydrothermal growth of ZnO nanowire arrays, ZnO nanoparticle (NP) seed was deposited on the FTO conductive glass by drop casting method. The synthesis of ZnO NP seed was modified from Pacholski method [29]. 10 mM zinc acetate dehydrate (Zn(CH_3_COO)_2_, Aldrich, Saint Louis, MO, USA) was fully dissolved in ethanol at 60 °C. 30 mM NaOH solution in ethanol was slowly added to the prior solution and mixed for 2 h at 60 °C. The synthesized ZnO NP seeds were 5–10 nm in diameter with spherical. After the ZnO seed layer deposition, the substrate was heated at 350 °C, which resulted in the uniform layer of ZnO nanocrystals. For the hydrothermal growth of ZnO nanowire, the seed-deposited substrate was immersed into an aqueous solution which containing of 50 mM zinc nitrate hexahydrate (Zn(NO_3_)_2_·6H_2_O, Aldrich), 25 mM hexamethylenetetramine (HMTA, C_6_H_12_N_4_, Aldrich), 5–7 mM polyethylenimine (PEI, C_2_H_5_N, Aldrich) and 0.35–0.45 M ammonium hydroxide (NH_4_OH, Aldrich) at 90–95 °C for 3–7 h. The residual polymer from the reaction was removed with ethanol washing and thermal annealing at 300 °C in air.

**Successive Photoreduction Process**—For the ZnO/CuO hetero nanostructure, copper sulfate (CuSO_4_) is added in deionized water for containing 1 M solution and a pristine ZnO nanowire arrays on the FTO glass is placed in the Cu^2+^ solution. Then, a high-powered UV lamp (Novascan, PSD Pro Series, Boone, IA, USA) was directly irradiated to the pristine ZnO for 1 min. Afterward, thermal annealing was practiced after 1st photoreduction process to form ZnO/CuO hetero nanostructure at 400 °C for 1 h. Meanwhile, silver nitrate (AgNO_3_) and gold chloride hydrate (HAuCl_4_) was dissolved in deionized water and prepared as 0.1 M metal precursor solutions. The ZnO/CuO hetero nanostructure was located on each solution and then UV lamp turned on to make metal nanoparticles, obtaining ZnO/CuO/M hierarchical nanostructure. The wavelength of the UV lamp approximately consisted of 185 nm (85%) and 254 nm (15%) with a power density of 2.5 W/cm^2^. All chemicals purchased in Sigma Aldrich. Different UV irradiation time was established as 10 min for silver nanoparticles and 3 min for gold nanoparticles. The diameter of the synthesized metal nanoparticles was approximately 5–10 nm [26].

**Characterizations**—The surface morphology and composition of the ZnO/CuO hetero nanostructure and the ZnO/CuO/M hierarchical nanostructure was observed with field-emission scanning electron microscopy (FE-SEM, Hitachi, S-4800, Seoul, Korea) and energy-dispersive X-ray spectroscopy (EDX, Seoul, Korea), respectively. The lattice structures of the material were determined by using transmission electron microscopy (Cs-TEM, Jeol, JEM-ARM200F, Seoul, Korea). The material phase was characterized with X-ray diffractometer (XRD, Rigaku, D/MAX-RC, Seoul, Korea). Optical properties were measured with UV-Vis spectroscopy (PerkinElmer, Lambda 650, Seoul, Korea).

**Photoelectrochemical Cell Measurement**—The PEC characteristic was measured by a potentiostat (Princeton Applied Research, VersaSTAT 3–450, Seoul, Korea). ZnO/CuO hetero nanostructure and ZnO/CuO/M hierarchical nanostructure was adopted as a working electrode. A Pt foil, Ag/AgCl electrode and 1 M sodium sulfate (Na_2_SO_4_) solution (pH: 6.2) were employed as a counter electrode, a reference electrode and an electrolyte, respectively. The light source was a 100 W metal halide lamp (Flosser, H1, Seoul, Korea) with an AM 1.5 G filter.

**FDTD simulation**—The FDTD simulation (Lumerical, Vancourver, BC, Canada) was conducted for the enhanced power absorption of the hierarchical nanostructure. The power absorbed mode was employed, where the wavelength varied from 300 nm to 1000 nm to cover the broad visible light region. The geometry of the nanostructure was designed as cylindrical ZnO/CuO nanostructure (~100/10 nm) and spherical metal nanoparticles (~10 nm). The scale of the geometry referred to the synthesized nanomaterials from SEM image. The optical properties of ZnO, CuO, Au and Ag from the data stored in the simulation program were used.

## 3. Results and Discussion

The ZnO/CuO/M hierarchical nanostructure is fabricated by a successive photoreduction process. The photoreduction process is basically a kind of photocatalytic synthesis, which reduced metal ions from the metal precursor solution along with electrons, generated within metal oxide nanostructure by external light energy. Therefore, this process can be expressed as:

*hv* (light energy) → e^−^ + h^+^ (within metal oxide nanostructure)
M^+^ + e^−^ (within aqueous solution) → M (metal nanoparticle)

**Figure 1:**
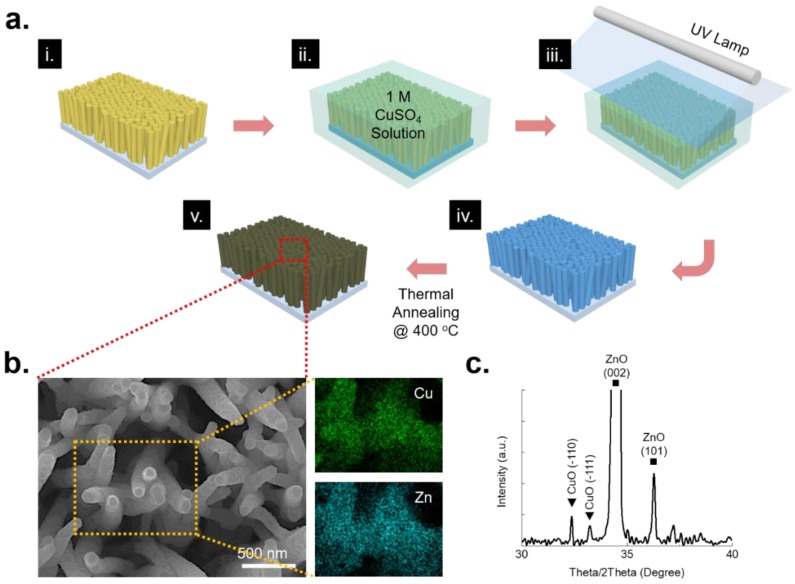
(**a**) A schematic of the fabrication process for ZnO/CuO hetero nanostructure; (**b**) Surface morphology of the ZnO/CuO hetero nanostructure and EDX component mapping images of Cu and Zn; (**c**) XRD spectra analysis of the ZnO/CuO hetero nanostructure, showing specific peaks of CuO and ZnO.

A fabrication of the hierarchical nanostructure begins from the synthesis of ZnO nanowire arrays on a FTO glass through hydrothermal growth. Hydrothermally grown ZnO nanowire arrays have needle-like shapes with 80–100 nm diameter and 10–12 µm length. Figure 1a represents the fabrication process for the ZnO/CuO hetero nanostructure. Prior to turning on the high intensity UV lamp for 1 min, the pristine ZnO nanowire was dipped into a 1 M of copper sulfate (CuSO_4_) solution. Since the color of a copper ion is blue, the solution looked blue. Afterward, the color of UV-treated ZnO nanowire is changed from light yellow to light blue and this indicates the copper ingredients, mostly amorphous copper, were coated on the ZnO nanostructure. The UV-treated ZnO nanowire was cleaned by ethanol and deionized water. Thermal annealing in a furnace at 400 °C heating for 1 h in air produces a ZnO/CuO hetero nanostructure [30]. At the same time, the color of the ZnO/CuO hetero nanostructure turns from light blue to black by complete CuO forming. As shown in Figure 1b, fabricated ZnO/CuO hetero nanostructure was examined by using SEM and EDX mapping, showing smooth surface and high uniformity of CuO layer. XRD analysis indicates clear evidence with specific peaks of the fabricated ZnO/CuO hetero nanostructure by a facile photoreduction process in Figure 1c.

**Figure 2:**
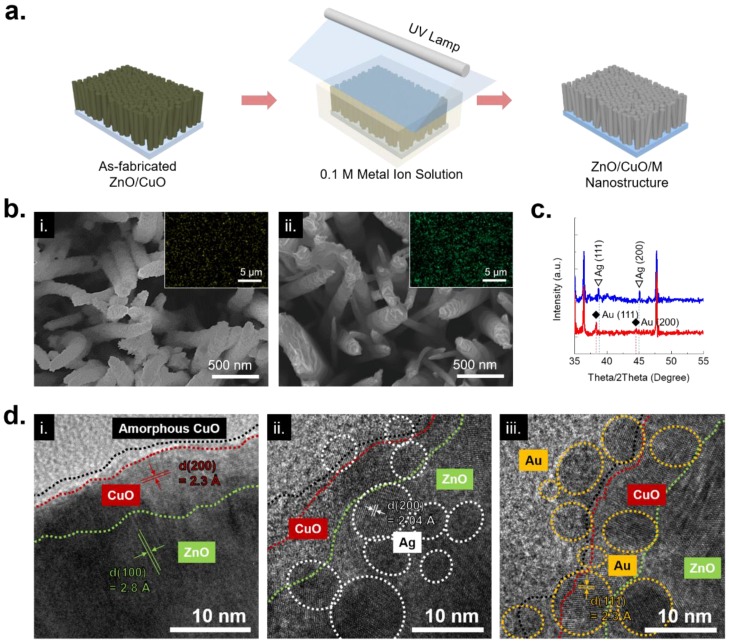
(**a**) A schematic of the successive photoreduction process for ZnO/CuO/M hierarchical nanostructure; (**b**) Surface images of synthesized (i) silver and (ii) gold nanoparticles on the ZnO/CuO hetero nanostructure. Insets are EDX mapping images; (**c**) XRD spectra analysis of the synthesized silver (blue line) and gold (red line) nanoparticles on the ZnO/CuO hetero nanostructure; (**d**) TEM images of (i) ZnO/CuO hetero nanostructure, (ii) ZnO/CuO/Ag and (iii) ZnO/CuO/Au hierarchical nanostructures.

For the additional metal nanoparticle formation, a successive photoreduction process was carried on the ZnO/CuO hetero nanostructure. Similar to the aforementioned photoreduction process, the ZnO/CuO hetero nanostructure is also immersed in a 0.1 M of metal ion solution. When UV lamp was turned on, metal ion started to turn into metal nanoparticles [25]. The synthesized metal nanoparticles are naturally located on the surface of the ZnO/CuO hetero nanostructure (Figure 2a). At this time, silver nitride (AgNO_3_) and gold chloride hydrate (HAuCl_4_) was used as a metal precursor for the synthesis of silver and gold nanoparticles respectively. In here, different UV irradiation condition was used on the silver (10 min) and gold nanoparticles (3 min). The digital images of the samples and metal precursors for the successive photoreduction process was represented in Figure S1. The synthesized metal nanoparticles on the ZnO/CuO nanostructure was observed by SEM in Figure 2b. The synthesized (i) silver nanoparticles entirely covered the surface of the ZnO/CuO hetero nanostructure. Likewise, the synthesized (ii) gold nanoparticles were placed on the ZnO/CuO hetero nanostructure. While the SEM analysis from silver and gold nanoparticles represents different surface morphology states, a high uniformity of the synthesized metal nanoparticles was observed in EDX mapping. Moreover, the synthesized silver and gold nanoparticles showed specific peaks by XRD analysis in Figure 2c. The TEM analysis measured lattice distances of the ZnO/CuO hetero nanostructure and ZnO/CuO/M hierarchical nanostructure in Figure 2d. The fabricated ZnO/CuO hetero nanostructure was shown in a core/shell structure, having lattice distance of (i) 2.8 Å for ZnO in (100) plane and 2.3 Å for CuO in (200) plane. In particular, a thin amorphous layer was observed and it might be one of the traces from the unclear copper ingredients after 1st photoreduction process and following thermal annealing. After successive photoreduction process, Ag and Au nanoparticles were individually formed onto the surface of the ZnO/CuO hetero nanostructure. The sizes of the synthesized metal nanoparticles were 5–10 nm, showing specific lattice distances of (ii) 2.04 Å for Ag in (200) plane and (iii) 2.3 Å for Au in (111) plane, respectively.

**Figure 3:**
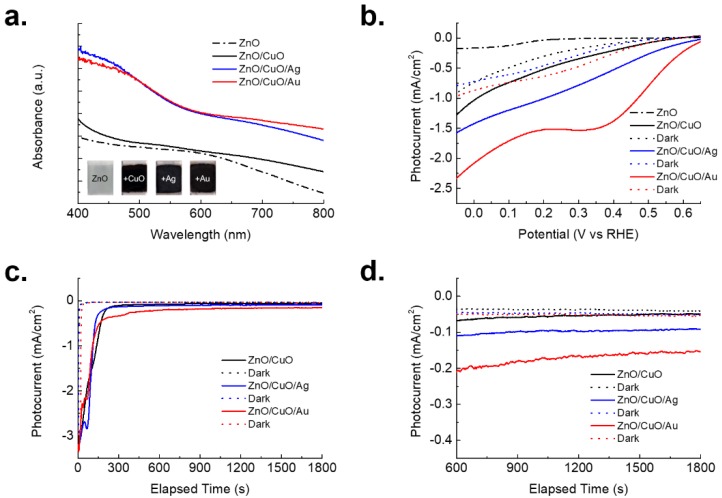
(**a**) Optical absorbance of the pristine ZnO, ZnO/CuO hetero nanostructure, ZnO/CuO/Ag hierarchical nanostructure and ZnO/CuO/Au hierarchical nanostructure. Inset shows actual digital images of the samples; (**b**) Linear voltammetry curves for the pristine ZnO, ZnO/CuO hetero nanostructures, ZnO/CuO/M hierarchical nanostructure and dark current; (**c**) Chronoamperometry analysis of the ZnO/CuO hetero nanostructures, ZnO/CuO/M hierarchical nanostructure and dark current during bias of 0.05 V vs. RHE; (**d**) Selected data for the chronoamperometry results from 600 s to the end. The output photocurrent is gradually saturated as −0.05 mA/cm^2^ for ZnO/CuO hetero nanostructure, −0.1 mA/cm^2^ for ZnO/CuO/Ag hierarchical nanostructure, −0.17 mA/cm^2^ for ZnO/CuO/Au hierarchical nanostructure while the dark currents are almost −0.03 mA/cm^2^.

The optical properties of the pristine ZnO, ZnO/CuO hetero nanostructure and ZnO/CuO/M hierarchical nanostructure were analyzed by UV-Vis spectroscopy. As shown in Figure 3a, an enhanced optical absorbance was measured from the all of the surface modified nanostructures, compared to pristine ZnO nanowire arrays. Especially, an absorbance intensity of ZnO/CuO/Ag hierarchical nanostructure was slightly larger than that of ZnO/CuO/Au hierarchical nanostructure at 400–500 nm while an opposite trend was obtained at 600–800 nm. This optical phenomenon was possibly originated from the different optical absorbance property of the Ag and Au nanoparticles. [31] Moreover, CuO possibly caused the unclear plasmon peaks from the Ag/Au nanoparticle. Typically, the maximum plasmon peaks of the Ag and Au nanoparticles are located at around 400 nm and 550 nm, respectively. While the Ag and Au nanoparticles were synthesized onto the ZnO/CuO hetero nanostructure, the major ingredients of the ZnO/CuO/M hierarchical nanostructure were still ZnO and CuO. Particularly, the CuO shell layer was completely covered on ZnO. Therefore, the absorbance curves were followed a trend of CuO. Inset images show actual digital images of the individual nanostructures before/after photoreduction process. In order to apply as a photocathode for the PEC cell, PEC property of ZnO/CuO/M hierarchical nanostructure is confirmed by using a three-electrode method. A linear voltammetry analysis showed unique photoelectrical properties over the ZnO/CuO, ZnO/CuO/Ag and ZnO/CuO/Au nanostructures, showing the metal nanoparticles contribute to have an enhanced output photocurrent possibly due to surface plasmon effect from the novel metal nanoparticles [32,33]. Depended on the physical property of the metal nanoparticle such as refractive index, particle size and distance, the synthesized gold nanoparticle on the ZnO/CuO hetero nanostructure showed better PEC performance than that of silver nanoparticles as shown in Figure 3b. After that, a chronoamperometry analysis was conducted to show output photocurrent stability at 0.05 V vs. RHE for 1800 s in Figure 3c. All of the output photocurrents from the ZnO/CuO, ZnO/CuO/Ag and ZnO/CuO/Au nanostructures was stabilized after 300 s. Figure 3d shows saturated output photocurrents from the various photocathodes. Similarly, silver and gold nanoparticles increased the output photocurrent, compared to the ZnO/CuO hetero nanostructure. Although ZnO/CuO/Ag hierarchical nanostructure showed slightly better optical absorbance property at 400–500 nm of wavelength than ZnO/CuO/Au hierarchical nanostructure, photoelectrochemical property and stability of Ag is normally weaker than that of Au nanoparticle. Therefore, ZnO/CuO/Au hierarchical nanostructure showed almost double output photocurrent than that of ZnO/CuO/Ag hierarchical nanostructure.

**Figure 4:**
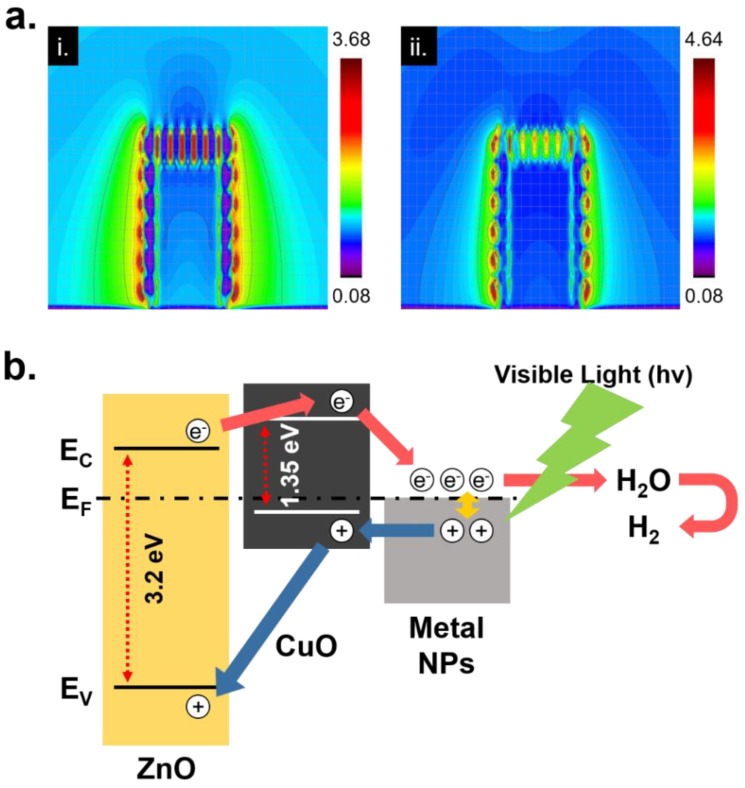
(**a**) FDTD simulation for showing generated surface plasmon enhancement from (i) silver and (ii) gold nanoparticles on the ZnO/CuO hetero nanostructure at the wavelength of 532 nm; (**b**) A schematic diagram of the theoretical bandgap structure at equilibrium state of the ZnO/CuO/M hierarchical nanostructure.

While the synthesized metal nanoparticles on the ZnO/CuO hetero nanostructure evidently provided an enhanced PEC property, surface plasmon effect differences, originated from the silver and gold nanoparticles was not clearly proven in the cell tests. As an alternative, a FDTD simulation was practiced to compare the surface plasmon effect from the silver and gold nanoparticles on the ZnO/CuO hetero nanostructure at a wavelength of 532 nm. Figure 4a represents the generated surface plasmon from the (i) silver and (ii) gold nanoparticles. A strong surface plasmon was observed from the gold nanoparticle at the same environments due to its higher refractive index in visible wavelength region [34] and better optical absorbance rate at a wavelength of 532 nm. To obtain highly enhanced plasmon effect, the size of target materials should be uniform shapes and well-ordered. However, in actual cases, the synthesized metal nanoparticles were occasionally aggregated with each other and randomly located onto the ZnO/CuO hetero nanostructure. Nevertheless, the metal nanoparticles contributed to enhanced electron generation by sunlight, as well as increased charge transfer efficiency. Figure 4b shows a theoretical band gap structure of the ZnO/CuO/M hierarchical nanostructure and hydrogen generation process when it was used as a photocathode. The ZnO/CuO hetero nanostructure showed inefficiency at equilibrium state due to their inherently improper bandgap structure [35,36]. However, a bias voltage changes bandgap structure and the metal nanoparticles on the ZnO/CuO hetero nanostructure would help to gain an enhanced PEC property by surface plasmon.

## 4. Conclusions

In summary, we proposed a wet-based, successive photoreduction process for the ZnO/CuO/M hierarchical nanostructure. The fabricated ZnO/CuO/Ag and ZnO/CuO/Au hierarchical nanostructure was measured and demonstrated by using various analytic tools. Consequently, the successive photoreduction process was proven to provide a facile surface modification method to the metal oxide nanostructure for PEC cell applications.

## Supplementary Materials

The following are available online at http://www.mdpi.com/2079-4991/8/5/323/s1.
