# Supplementary material for: ZnO/CuO/M (M = Ag, Au) Hierarchical Nanostructure by Successive Photoreduction Process for Solar Hydrogen Generation
Source: Nanomaterials (Basel). 2018 May 12;8(5):323. doi: 10.3390/nano8050323 (PMC5977337; doi:10.3390/nano8050323)
Supplement: Supplementary file 1 [file nanomaterials-08-00323-s001.pdf]

Supplementary Materials

ZnO/CuO/M (M = Ag, Au) Hierarchical Nanostructure by Successive Photoreduction Process for Solar Hydrogen Generation

Jinhyeong Kwon [1,†], Hyunmin Cho [1,†], Jinwook Jung [1], Habeom Lee [1], Sukjoon Hong [2], Junyeob Yeo [3], Seungyong Han [4,*] and Seung Hwan Ko [1,5,*]

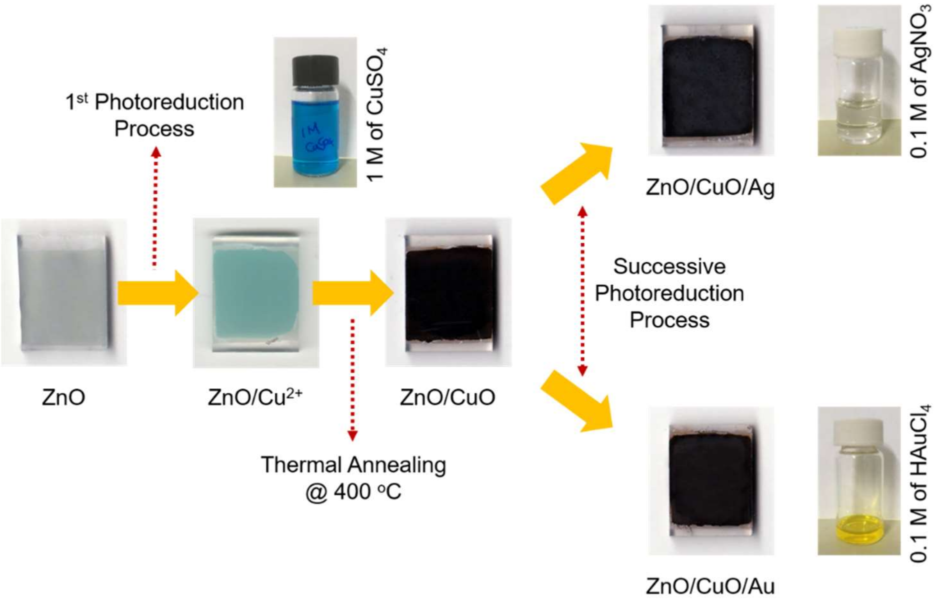

Figure S1. Digital images of samples and metal precursors of the successive photoreduction process
